# Thermodynamics and Statistical Mechanics of Small Systems

**DOI:** 10.3390/e20060392

**Published:** 2018-05-23

**Authors:** Andrea Puglisi, Alessandro Sarracino, Angelo Vulpiani

**Affiliations:** 1 Consiglio Nazionale delle Ricerche (CNR), Istituto dei Sistemi Complessi (ISC), c/o Dipartimento di Fisica, Universita’ Sapienza Roma, p.le A. Moro 2, 00185 Roma, Italy; 2 Dipartimento di Fisica, Università degli studi di Roma “La Sapienza”, Piazzale A. Moro 5, 00185 Roma, Italy; 3 Centro Interdisciplinare “B. Segre”, Accademia dei Lincei, 00100 Roma, Italy

**Keywords:** statistical mechanics, small systems, stochastic thermodynamics, non-equilibrium fluctuations, large deviations

A challenging frontier in modern statistical physics is concerned with systems with a small number of degrees of freedom, far from the thermodynamic limit. Beyond the general interest in the foundation of statistical mechanics, the relevance of this subject is due to the recent increase of resolution in the observation and in the manipulation of biological and man-made objects at micro- and nano-scales. The peculiar feature of small systems is the role played by fluctuations, which cannot be neglected and are responsible for many non-trivial behaviors. The study of fluctuations of thermodynamic quantities, such as energy or entropy, goes back to Einstein, Onsager, and Kubo; more recently, interest in this matter has grown with the establishment of new fluctuation–dissipation relations, which hold even in non-linear regimes, and of the so-called stochastic thermodynamics. Such a turning point has received a great impulse from the study of systems that are far from thermodynamic equilibrium, due to very long relaxation times, as in disordered systems, or due to the presence of external forcing and dissipation, as in granular or active matter. Applications of the thermodynamics and statistical mechanics of small systems range from molecular biology to micro-mechanics, including, among others, models of nano-transport, of Brownian motors, and of (living or artificial) self-propelled organisms.

## The Contributions

In this special issue, we collect 20 contributions, spanning the above mentioned subjects. In particular, the main addressed topics are as follows:

1. entropy production and stochastic thermodynamics (8);
2. heat transport and entropy in nonlinear chains and long-range systems (4);
3. granular and other dissipative systems (2);
4. phase transitions and large deviations in probabilistic models (2);
5. coarse-graining techniques (2);
6. ferromagnetic models (2).

## Topic (1): Entropy Production and Stochastic Thermodynamics

In [1], the stochastic energetics approach is applied to a Stirling engine model. Such an engine is realized by coupling a passive and an active system. The latter is modeled through a non-Gaussian noise with persistency: this choice allows the authors to study two possible origins of discrepancy with respect to thermodynamic engines (coupled to equilibrium systems), finding that persistency is more important.

In [2], the interesting problem of optimizing the energetic cost of maintaining a non-equilibrium state is discussed. The important result concerns the existence of a minimum bounding such a cost, which is expressed as an information-theoretic measure of distinguishability between the target non-equilibrium state and the underlying equilibrium distribution.

In [3], the possibility of a Clausius relation for active systems is investigated on the basis of the so-called Active Ornstein-Uhlenbeck Particles (AOUP) model. It is shown that a mapping from the AOUP model to an underdamped model with non-uniform viscosity and temperature can shed light on this question, but induces an ambiguity in the determination of the parity under time-reversal of some forces in the system, leaving at least two possible definitions of entropy production. One of the two possible choices leads to an entropy production that is consistent with detailed balance in the system and can be expressed in a Clausius-like fashion.

In [4], the problem of moving a system, in a finite time, from an equilibrium state to a different one is studied. The question here is when such a transformation occurs optimally in the sense of producing a minimum amount of entropy. The answer is a set of constraints on the possible protocols, particularly on their time-derivatives. Some interesting examples related to recent experiments are discussed.

In [5], the author revisits the classical problem of a system in contact with a collection of harmonic oscillators initially in thermal equilibrium, which may represent a thermal bath. The new ingredient is a time-dependent system-bath coupling which is shown to lead to an additional harmonic force acting on the system. The consequences for heat and work functionals of stochastic thermodynamics, in classical as well as quantum systems, are also worked out.

In [6], a magnetic quantum thermal engine is considered where the energy levels are degenerate. The analytical expression of the relation between the magnetic field and temperature along the adiabatic process is calculated, including the efficiency as a function of the compression ratio.

In [7], two interacting stochastic systems are considered, under the point of view of information exchange. An information landscape and an information flux are defined and seen to influence different aspects of the systems’ dynamics. Connections with the entropy production of non-equilibrium thermodynamics are investigated. 

In [8], a stochastic model of nanopore is investigated in the framework of information dynamics, focusing on the local and specific entropy rates computed in simulations. Those metrics are put in relation with the fluctuations of the current in the nanopore.

## Topic (2): Heat Transport and Entropy in Nonlinear Chains and Long-Range Systems 

In [9], transport of mass and energy through a discrete nonlinear Schrödinger chain in contact with a heat reservoir and a pure dissipator is considered. Depending on the heat bath temperature, two interesting regimes are observed, featuring a non-monotonous shape of the temperature profiles across the chain (at low temperature) and a spontaneous emergence of discrete breathers (at high temperature), whose statistics can be described in terms of large deviations.

In [10], a multi-partitioned piston model coupled with two thermal baths at different temperatures is discussed in the framework of kinetic theory, obtaining the values of the main thermodynamic quantities characterizing the stationary non-equilibrium states: a good agreement with Fourier’s law in the thermodynamic limit is obtained. 

In [11], the effect of both short- and long-range interparticle interactions in the Nosé–Hoover dynamics of many-body Hamiltonian systems is investigated. It was found that the equilibrium properties of the system coincides with that within the canonical ensemble; however, in the case with only long-range interactions, the momentum distribution relaxes to its Gaussian form in equilibrium over a scale that diverges with the system size. This study brings to the fore the crucial role that interactions play in deciding the equivalence between Nosé–Hoover and canonical equilibrium.

In [12], the relation between Kolmogorov entropy and the largest Lyapunov exponent in systems with long-range interactions is studied. In particular, Lyapunov spectra for Coulombic and gravitational versions of the one-dimensional systems of parallel sheets with periodic boundary conditions are computed, showing that the largest Lyapunov exponent can be viewed as a precursor of the transition that becomes more pronounced as the system size increases.

## Topic (3): Granular and Other Dissipative Systems

In [13], the interesting issue of Kovacs-like memory effects, which characterize some athermal dissipative systems whose stationary states cannot be completely characterized by macroscopic variables such as pressure, volume, and temperature, is addressed. Within the linear response regime, it is proved that the observed non-monotonic relaxation is consistent with the monotonic decay of the non-equilibrium entropy.

In [14], the transport properties of a low-density granular gas immersed in an active fluid, modeled as a non-uniform stochastic thermostat, are investigated. Navier–Stokes hydrodynamic equations can describe the steady flow in the system, even for high inelasticity.

## Topic (4): Phase Transitions and Large Deviations in Probabilistic Models

In [15], an example of the condensation of fluctuations is considered in probabilistic models with power-law distributions. This kind of phenomenon occurs when there is a critical threshold above which the fluctuation in the system is fed by just one degree of freedom. The paper focuses on the evaluation of the participation ratio as a generic indicator of condensation.

In [16], a stochastic logistic model with multiplicative noise, which shows a transition for sufficiently strong noise, is studied. Such a transition between different solutions is analyzed in terms of entropy and information length.

## Topic (5): Coarse-Graining Techniques

In [17], a systematic coarse-graining methodology to treat many particle molecular systems using cluster expansion techniques is discussed. This allows for the building of effective Hamiltonians with interaction potentials with two, three, (and more) body interactions.

In [18], spatial block analysis as a method of efficient extrapolation of thermodynamic quantities from finite-size computer simulations of a large variety of physical systems is reviewed. Such a method provides promising results for simple liquids and liquid mixtures.

## Topic (6): Ferromagnetic Models

In [19], the authors focus on the Ising model with a small number of sites, comparing numerical results about the magnetization (in 1d, 2d, and 3d and without periodic boundary conditions) with past results for the thermodynamic limit.

In [20], the spinor analysis is applied to exactly evaluate spin–spin correlation functions in the 2d rectangular Ising model. The author shows the different results (even in terms of short- or long-range order) emerging from different boundary conditions.
